# Analysis of Risk Factors Associated with Proximal Junctional Kyphosis Following Long Instrumented Fusion from L1 to Sacrum: Age Itself Does Not Independently Increase the Risk

**DOI:** 10.3390/medicina60091441

**Published:** 2024-09-03

**Authors:** Joonghyun Ahn, Young-Hoon Kim, Yong-Chan Kim, Ki-Tack Kim, Sung-Min Kim, Jun Bum Park, Kee-Yong Ha

**Affiliations:** 1 Department of Orthopedic Surgery, Bucheon St. Mary’s Hospital, College of Medicine, The Catholic University of Korea, Bucheon 14647, Republic of Korea; ajhssnim@gmail.com; 2 Department of Orthopedic Surgery, Seoul St. Mary’s Hospital, College of Medicine, The Catholic University of Korea, Seoul 06591, Republic of Korea; boscoa@catholic.ac.kr; 3 Department of Orthopaedic Surgery, Kyung Hee University Hospital at Gangdong, College of Medicine, Kyung Hee University, Seoul 02447, Republic of Korea; yckimspine@gmail.com (Y.-C.K.); ktkim713@gmail.com (K.-T.K.); osdrksm83@gmail.com (S.-M.K.); tomjb@naver.com (J.B.P.)

**Keywords:** thoracolumbar junction, L1 vertebra, proximal junctional kyphosis, risk factor, instrumented fusion

## Abstract

*Background and Objectives:* This study is a retrospective analysis aimed at understanding the incidence and risk factors of proximal junctional kyphosis (PJK) following long-instrumented spinal fusion from L1 to the sacrum in patients with mild to moderate sagittal imbalance. *Materials and Methods:* It recruited consecutive patients undergoing instrumented fusion from L1 to the sacrum for degenerative lumbar disease between June 2006 and November 2019 in a single institution. The patients’ preoperative clinical data, muscle status at T12-L1 on magnetic resonance images, and sagittal spinopelvic parameters were analyzed. Univariate analysis was used to compare clinical and radiographic data between PJK and non-PJK patients. Logistic regression analysis was used to investigate the independent risk factors for PJK. *Results:* A total of 56 patients were included in this study. The mean age at surgery was 67.3 years and mean follow-up period was 37.3 months. In total, 10 were male and 46 were female. PJK developed in 23 (41.1%) out of 56; of these patients, 20 (87.0%) developed PJK within 1 year postoperatively. In the univariate analysis between PJK and non-PJK patients, the PJK group showed more frequent osteoporosis, lower body mass index, smaller cross-sectional area (CSA) and more fat infiltration (FI) in erector spinae muscle at T12-L1 and larger preoperative TLK and PT with statistical significance (*p* < 0.05). In the logistic regression analysis, severe (>50%) FI in erector spinae muscle (OR = 43.60, CI 4.10–463.06, R_2_N = 0.730, *p* = 0.002) and osteoporosis (OR = 20.49, CI 1.58–264.99, R_2_N = 0.730, *p* = 0.021) were statistically significant. *Conclusions:* Preexisting severe (>50%) fat infiltration in the erector spinae muscle and osteoporosis were independent risk factors associated with PJK following instrumented fusion from L1 to the sacrum, but age was not a risk factor.

## 1. Introduction

Since long spinal fusion for degenerative lumbar disease can theoretically increase biomechanical stress along with natural degeneration on the adjacent segments, a problematic complication called proximal junctional failures (PJF) may occur more or less postoperatively. Incidence of PJF and various potential risk factors associated with PJF have been reported and stratified in the literature [1,2,3,4,5,6,7,8,9,10]. Among them, there have been consistent reports opposed to the selection of upper instrumented vertebra (UIV) at the thoracolumbar junction in long-instrumented spinal fusion [5,10,11]. Kim et al. [10] previously investigated surgical outcomes of long spinal fusion based on proximal fusion level for adult spinal deformity. They reported high rate of sagittal imbalance, proximal adjacent segmental problems (proximal disc degeneration with kyphosis, compression fracture and screw failure at UIV) in proximal fusion up to L1 or L2, and concluded that instrumented lumbosacral fusions with UIV at L1 or L2 could not be recommended. This was understood due to the fact that the thoracolumbar junction was a transitional zone from the less-flexible thoracic spine to the flexible lumbar spine, and Kim et al. [10] explained concretely that stopping at or distal to T11 and T12 without costosternal articulation could not have a biomechanical advantage of thoracic cage on the adjacent segment in correction surgery for adult spinal deformity (ASD). However, there are some recent studies with an opposing point of view regarding proximal adjacent segment degeneration after long spinal fusion [4,12,13]. Bess et al. [13] demonstrated that the distribution of forces across and supra-adjacent to the UIV via posterior polyester tethers could allay the risk of PJF, which could potentially mitigate the risk of PJF. Other studies claim that the thoracolumbar junction could be considered selectively as UIV without certain potential risk factors [4,12].

Although the determination of UIV has been a controversial issue in long spinal instrumented fusion for correction of ASD [11,12,14,15], which still has no definite answer up to date. To the best of our knowledge, there have been a paucity of studies investigating the risk factors associated with PJF following long-instrumented spinal fusion from L1 to sacrum. The purpose of this study was to investigate the incidence and the risk factors of PJF following long-instrumented fusion from L1 to sacrum and to shed new light on L1 vertebra as UIV for degenerative lumbar disease with mild to moderate sagittal imbalance.

## 2. Materials and Methods

### 2.1. Study Design and Patient Population

After obtaining approval by the appropriate ethics committee (institutional review board of our institution), a retrospective review of clinical and radiographic data were performed. This study was performed with degenerative lumbar disease patients who had undergone instrumented spinal fusion between March 2006 and December 2019 at a single institution.

**Figure 1:**
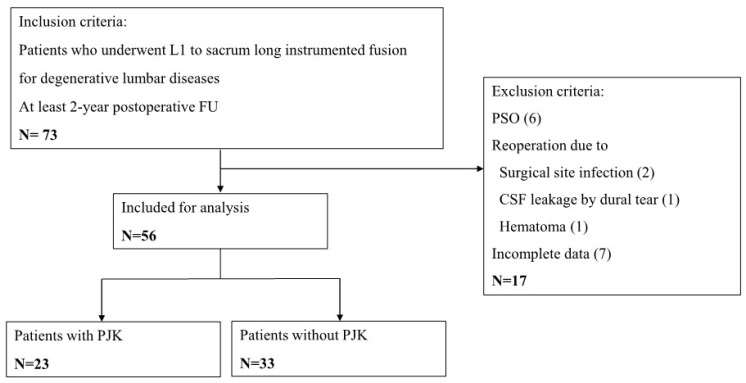
Flowchart of patient selection for analysis. Period of inclusion was 2006 to 2019. CSF, cerebrospinal fluid; PSO, pedicle subtraction osteotomy; FU, follow-up.

The inclusion criteria were as follows: (1) preoperative diagnosis of degenerative lumbar diseases (spinal stenosis, spondylolisthesis, adjacent segment disease following previous spinal fusion, lumbar degenerative kyphosis); (2) completion of a long-segment spinal instrumented fusion surgery from L1 vertebra to the sacrum regardless of iliac fixation; and (3) a postoperative follow-up period of at least 2 years. The exclusion criteria were as follows: (1) sagittal balance as C7 sagittal vertical axis (C7SVA) more than 15 cm on lateral whole spine radiograph in standing position; (2) inadequate visibility for measuring radiographic parameters in whole spine standing lateral radiographs at regular pre- and postoperative visits; (3) history of pedicle subtraction osteotomy (PSO) or other equivalent procedures; (4) postoperative surgical site infection requiring revisional operation for index surgery, but not for PJF; and (5) if they lacked either baseline or postoperative imaging at regular (preoperative, postoperative 3 months, 1-year and 2-year) follow-up (Figure 1).

### 2.2. Data Collection

**Figure 2:**
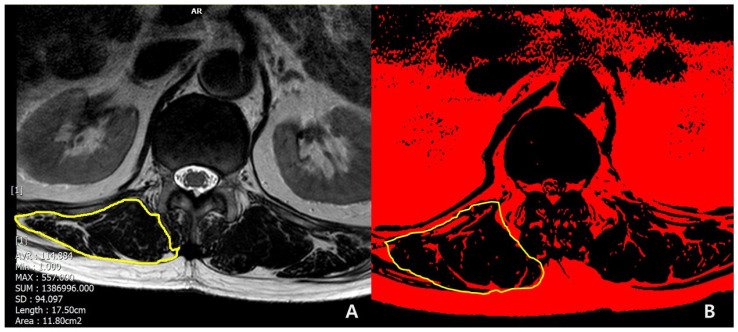
Methods for measuring (**A**) CSA and (**B**) FI of erector spinae muscle.

The following demographic and surgical data were collected: age at surgery; sex (female/male); bone mineral density (BMD, T-score); body mass index (BMI, kg/m^2^); smoking; previous spinal fusion; American Society of Anesthesiologist physical status (ASA) grading; iliac fixation; disc degeneration by Pfirrmann grade [16] at T12-L1 segment (1,2,3: mild to moderate; 4,5: severe); muscularity status at T12-L1 (cross-sectional area [CSA], cm^2^); and fat infiltration grade (mild to moderate, if <50%; severe, if ≥50%) in erector spinae muscle at T12-L1 in T2-weighted axial magnetic resonance (MR) images using Picture Archiving and Communication System (PACS) by Philips ACHIEVA 3.0T (Philips Healthcare, West Sussex, UK) and the ImageJ for windows (National Institutes of Health, Bethesda, MD, USA, V 1.8.0). The region of interest (ROI) for erector spinae muscle at the T12-L1 level was outlined polygonally with the manipulation of a graphic cursor and was handled with the pseudocoloring method [17] (Figure 2). Then, CSAs (Figure 2A) were calculated, and the fat infiltration grade (Figure 2B) was determined.

**Figure 3:**
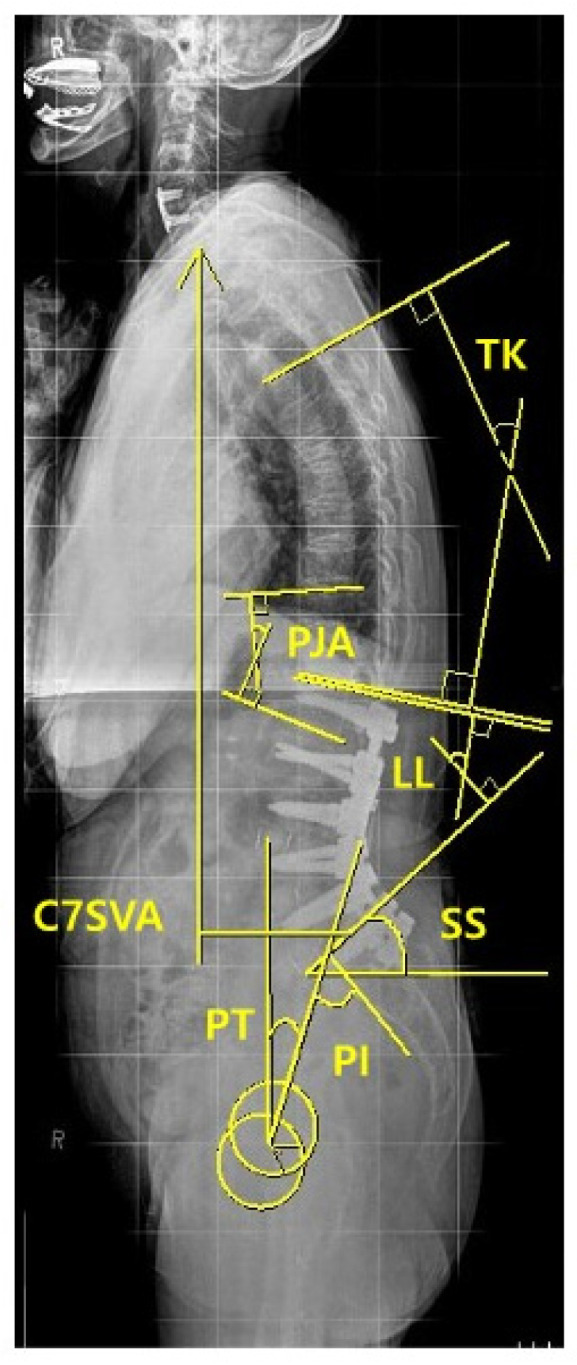
Measurement of sagittal spinopelvic parameters.

For the radiographic assessment of spinopelvic parameters, standing 36-inch-long cassette AP and lateral radiographs of the whole spine were measured at the preoperative, immediate postoperative, and regular postoperative outpatient visits (3, 6, 9 months, and 1-year, 2-year, and the final follow-up), respectively. On the radiographs, C7 sagittal vertical axis (C7SVA), thoracic kyphosis (TK) [18], thoracolumbar kyphosis (TLK) [18,19], lumbar lordosis (LL) [18], sacral slope (SS) [18], pelvic tilt (PT) [18], pelvic incidence (PI) [18,19,20], and proximal junctional angle (PJA) were measured (Figure 3). We also collected and analyzed the change (preoperative minus postoperative) of each radiographic parameter between the preoperative and the immediate (3 months) postoperative.

## 3. Results

### 3.1. Baseline Demographics and PJK Incidence of Included Patients

Among the 73 consecutively treated degenerative lumbar disease patients with completion of instrumented spinal fusion from L1 to the sacrum, a total of 56 patients met the study inclusion criteria and were recruited as the study cohort. There were 46 women (82.1%) and 10 men (17.9%). The age of patients was 67.3 ± 7.9 (range, 45–79) years, and BMI was 26.1 ± 3.9 (range, 19.3–40.1) kg/m^2^. There were 18 (32.1%) osteoporotic patients. Iliac fixation was supplemented in 30 (53.6%) patients. Eighteen (32.1%) patients had a previous history of spinal fusion. Through the total follow-up period, PJK were diagnosed in 23 (41.1%) out of 56 patients. Of these, 20 (87.0%) were observed within 1 year postoperatively. The demographics data and incidence of PJK are further summarized in Table 1 and Table 2.

### 3.2. Univariate Analysis of Demographic or Surgical Risk Factors

**Figure 4:**
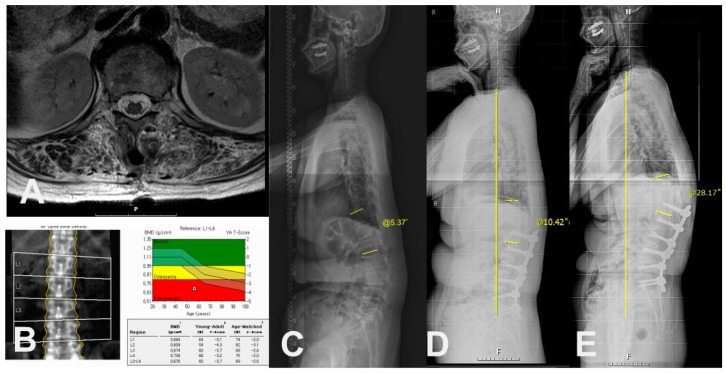
Case of 66-year-old female patient with PJK. (**A**) Preoperative axial T2-weighted magnetic resonance image showing erector spinae muscle with severe (>50%) fat infiltration at T12-L1 level. (**B**) Preoperative BMD showing osteoporosis. (**C**) Preoperative whole spine standing lateral radiograph showing PJA of 5.37°. (**D**) Postoperative 3-month standing lateral whole spine radiograph showing PJA of 10.42°. (**E**) Postoperative 2-year standing lateral whole spine radiograph showing PJK (PJA of 28.17°) with increased C7SVA.

**Figure 5:**
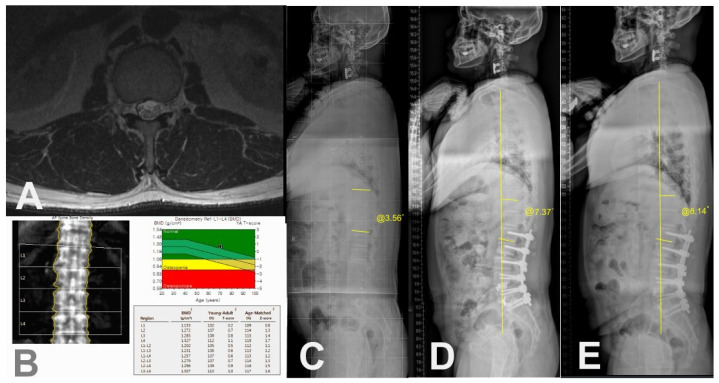
A case of a 73-year-old female patient without PJK. (**A**) Preoperative axial T2-weighted magnetic resonance image showing erector spinae muscle with minimal fat infiltration at T12-L1 level. (**B**) Preoperative BMD showing no osteoporosis. (**C**) Preoperative whole spine standing lateral radiograph showing PJA of 3.56°. (**D**) Postoperative 3-month whole spine standing lateral radiograph showing PJA of 7.37°. (**E**) Postoperative 3-year standing lateral whole spine radiograph showing PJA of 8.14° with maintained C7SVA.

In a univariate analysis of demographics, PJK group showed more frequently in females (100% in PJK vs. 73% in non-PJK, *p* = 0.011) and osteoporosis (60.9% in PJK vs. 12.1% in non-PJK, *p* < 0.001). Also, the PJK group showed the lower mean value of the lowest T-score in BMD (−2.4 ± 0.8 in PJK vs. −1.5 ± 1.1 in non-PJK, *p* = 0.006) and BMI (24.4 ± 2.4 in PJK vs. 27.1 ± 4.3 in non-PJK, *p* = 0.004) with statistical significance. In a comparison of muscular quantity between the groups, there were smaller CSAs (cm^2^, 23.8 ± 4.2 vs. 32.2 ± 10.1, *p* < 0.001) in erector spinae muscles at T12-L1 in PJK group than the non-PJK group. Also, in a comparison of muscular quality between the groups, PJK group showed more frequent severe (≥50%) fat infiltration in erector spinae muscles at T12-L1 with statistical significance (*p* < 0.001) (Table 3 and Figure 4 and Figure 5).

There were no significant differences in age at surgery, ASA grade, previous spinal fusion, smoking, iliac fixation, and disc degeneration status at T12-L1 between the groups. The follow-up period (months) was different (61.1 ± 40.6 in PJK vs. −23.6 ± 24.2 in non-PJK, *p* < 0.001) (Table 3).

### 3.3. Univariate Analysis of Radiographic Risk Factors after L1-Sacrum Instrumented Fusion

In a comparison of preoperative radiographic parameters between the groups, PT (°) was larger in the PJK group than the non-PJK group (34.0 ± 7.2 vs. 25.8 ± 13.0, *p* = 0.032). There were no significant differences in preoperative TK, TLK, LL, SS, PI, C7SVA, and PJA (*p* > 0.05).

In comparison of the immediate (3 months) postoperative radiographic parameters, the PJK group showed larger immediate postoperative TLK (23.5 ± 9.9 vs. 8.7 ± 12.2, *p* < 0.001) and PJA (20.9 ± 7.7 vs. 10.7 ± 8.1, *p* < 0.001), respectively. However, there were no differences in the other immediate postoperative parameters (*p* >0.05).

Although the changed amount of LL was larger in the PJK group, there was no statistical difference (36.6 ± 27.7 vs. 24.8 ± 27.2, *p* = 0.180). There was no significant difference in the changes in other radiographic parameters from the preoperative to the immediate postoperative values between the two groups (Table 4).

### 3.4. Logistic Regression Analysis of Potential Risk Factors Associated with PJK after L1-Sacrum Instrumented Fusion

The variables with a *p*-value <0.05 in Table 2 and Table 3 were selected after univariate analyses and were analyzed further with a logistic regression analysis. The logistic regression analysis revealed that severe (>50%) fat infiltration in erector spinae muscle (OR = 43.60, CI 4.10–463.06, R_2_N 0.730, *p* = 0.002) and osteoporosis (OR = 20.49, CI 1.58–264.99, R_2_N 0.730, *p* = 0.021) were statistically significant (Table 5). Female sex (*p* = 0.167), BMI (*p* = 0.620), axial CSA of erector spinae muscle at T12-L1 (*p* = 0.082), preoperative PT (*p* = 0.852), immediate postoperative TLK (*p* = 0.161), and PJA (*p* = 0.124) were not statistically significant (Table 5).

## 4. Discussion

Causes of PJK following long-instrumented spinal fusion can be explained with problems of soft tissue (intervertebral disc, paravertebral muscle, posterior ligamentous complex, et cetera.) and/or of bony structures (UIV or UIV + 1 fracture, loss of mechanical stability between instruments and bony vertebra). According to the published systematic review articles [21,22], age, female gender, and low BMD/osteoporosis were demographic risk factors for PJK. The current study revealed that osteoporosis and severe (>50%) fat infiltration in erector spinae muscle at the proximal junction (T12-L1 level) were independent risk factors for PJK following instrumented fusion from L1 to the sacrum, but age was not a risk factor. Therefore, if a surgeon plans to operate patients with instrumented fusion from L1 to the sacrum, we suggest that patients with osteoporosis and severe fat infiltration in erector spinae muscle at T12-L1 should be avoided or treated with a different surgical plan. Moreover, in patients who maintained quality in the erector spinae muscle, the L1 vertebra as a UIV can be applied during fusion surgery regardless of age.

Interestingly, it was observed that most of PJK (88%) developed as a result of bony structural problems such as adjacent vertebral compression fractures. Ha et al. [14] reported bony failures, such as compression fractures at the proximal junction, were more prevalent in the patients with distal thoracic vertebra as a UIV, whereas subluxation was more prevalent in the patients with proximal thoracic vertebra as a UIV. The observed outcome that fractures at the TLJ were the main cause of PJK was considered a quite understandable result in that it suggests that osteoporosis is a condition with a high risk of fracture [23] and that the location is mainly at the TLJ. On the other hand, the degeneration of the T12-L1 intervertebral disc, which is the boundary of the thoracic and lumbar spine, was not a significant risk factor of PJK, which is believed to be due to the stabilization of the adjacent segment to the fusion as the degeneration of the intervertebral disc. This is in line with the concept mentioned in the past literature that the degeneration process of the intervertebral disc could be subdivided into three stages: (1) temporary dysfunction; (2) unstable phase; and (3) stabilization [24]. Based on the above, if fusion for the elderly patient is stopped at the thoracolumbar junction, it is considered that the problem of bony structure would be higher than that of soft tissue.

Re-operations were performed early after index surgery in the cases of PJK (PJF) accompanied by severe clinical symptoms due to screw loosening or adjacent vertebral fracture. According to the criteria for exclusion from this study, therefore, they were excluded from statistical analysis because of reoperation within 1 year.

As an analysis of muscle quantity (CSA) and quality (FI), only severe (>50%) FI was found to be a significant risk factor. Muscle size may vary depending on the patient’s body size, as fat infiltration itself is associated with ageing, meaning that muscle function has decreased, and thus is thought to adversely affect maintaining dynamic spinal stability.

Age did not reveal any statistical significance in this study, which can predict that limited fusion with L1 as UIV can be performed regardless of age for patients who maintain the state of the musculoskeletal system, because functional age representing bone density or muscle conditions differs from chronological age itself, which can be improved through resistance exercise [25].

The current study showed that LL, PT, and SVA before surgery were not significant risk factors in the logistic regression model, which was different from previous studies that reported that a large amount of correction was a risk factor for PJK [26,27,28,29]. In this study, it is believed that the amount of correction was not enough to cause PJK even in patients who received relatively large corrections, since PSO or the other equivalent procedure was not performed.

In terms of surgical risk factors, it was reported that using hooks at the UIV and the selection of the UIV above T8 could reduce the occurrence of PJK, while pelvic fixation was significantly associated with increased occurrence of PJK [21,22]. Pelvic fixation is thought to increase PJK in addition to the known risk factor of proximal fixation segment at the thoracolumbar junction (L1 vertebra) in the patients of this study.

Whereas several studies defined PJK as being (1) PJA ≥ 10° and (2) 10° greater than the preoperative measurement [14,30], we used (1) PJA ≥ 20° as a proposed critical value of PJK in the current study. There were two reasons for this cut-off value of PJK. First, 10° or 15° of PJK in the literature failed to demonstrate such a critical angle in terms of clinical outcomes; Bridwell et al. evaluated “20” degrees as a critical angle to diagnose PJK [31]. However, even with ≥20° as a critical angle for PJK, there were not any significant clinical differences in the pre- and postoperative ODI, but only with the trend between the PJK group and the non-PJK group.

This study has several limitations. It is a retrospective study with a relatively small number of patients. However, it indicates the risk factors which had statistically significant association with the PJK, despite the limited sample size. To clarify the significance of these variables, replication studies and future multicenter research with a larger number of participants is recommended to help validate and strengthen the findings. It would also be helpful to utilize artificial intelligence in dealing with a larger number of participants [32].

In addition, most of the PJKs occurred relatively early (within 1 year postoperatively) in the current study. It is thought that the meaning of PJK occurring early after surgery and after a long period of time would be different. In this regard, it is also necessary to conduct research with a long follow-up period. Moreover, we could not investigate surgical factors potentially affecting early failures, such as graft materials for fusion, improper screw placement at the UIV, and lordosis distribution index.

## 5. Conclusions

This study found severe (≥50%) fat infiltration in erector spinae muscles at T12-L1, and osteoporosis were independent risk factors for PJK in patients undergoing L1-sacrum instrumented fusion. To avoid early-onset PJK, consideration of these factors is important before planning long-instrumented spinal fusion from L1 to the sacrum for patients with degenerative lumbar disease. However, since age itself is not a risk factor, L1 can be selected as UIV even in elderly patients if there are no muscle-related risk factors and osteoporosis.

## Figures and Tables

**Table 1:** Baseline demographics of included patients treated with instrumented spinal fusion from L1 to the sacrum.

Variables	Values
No. of included patients	56
Age at surgery (years)	67.3 ± 7.9 (range, 45–79)
Sex	Female 46; Male 10
BMI (kg/m^2^)	26.1 ± 3.9 (range, 19.3–40.1)
BMD (T-score)	−1.8 ± 1.1 (range, −3.50–1.40)
Osteoporosis, *n* (%)	18 (32.1)
Follow-up period (months)	43.5 ± 31.7 (range, 24–136)
Smoker, *n* (%)	7 (12.5)
ASA grade	
I, *n* (%)	9 (16.1)
II, *n* (%)	39 (69.6)
III, *n* (%)	8 (14.3)
Iliac screw fixation, *n* (%)	30 (53.6)
Previous spinal fusion, *n* (%)	18 (32.1)

Values are presented as mean ± standard deviation with range or number(s) (with or without percentages). BMI, body mass index; BMD, bone mineral density; ASA, American Society of Anesthesiologists.

**Table 2:** Incidence and reasons of PJK in included patients treated with instrumented spinal fusion from L1 to the sacrum.

Incidence of PJK Until Final Follow-Up, *n*/Total (%)	23/56 (41.1)
PJK within postoperative 1 year out of total PJK, *n*/total PJK (%)	20/23 (87.0)
Compression fracture, *n*/total PJK (%)	17/23 (73.9)
Screw loosening/pull-out, *n*/total PJK (%)	4/23 (17.4)
Screw loosening with L1 upper endplate penetration, *n*/total PJK (%)	2/23 (8.7)
Instability or subluxation of proximal adjacent segment, *n*/total PJK (%)	3/23 (13.0)
PJF within PJK	6/23 (26.1)

Values are presented as mean ± standard deviation with range or number(s) (with or without percentages). PJK, proximal junctional kyphosis; PJF, proximal junctional failure.

**Table 3:** Univariate analysis of demographic and surgical risk factors.

Parameters	PJK Group (*n* = 23)	Non-PJK Group (*n* = 33)	*p*
Age (years)	66.4 ± 5.3	67.7 ± 9.2	0.134
Female sex, *n* (%)	23 (100)	23 (72.7)	0.011 *
BMD (T-score)	−2.4 ± 0.8	−1.5 ± 1.1	0.006 *
Osteoporosis, *n* (%)	14 (60.9)	4 (12.1)	<0.001 *
BMI (kg/m^2^)	24.4 ± 2.4	27.1 ± 4.3	0.004 *
Follow-up period (months)	61.1 ± 40.6	23.6 ± 24.2	<0.001 *
ASA grade			0.970
Grade I, *n* (%)	6	8	
Grade II, *n* (%)	14	20	
Grade III, *n* (%)	3	5	
Smoking, *n* (%)	2 (8.7)	5 (15.2)	0.758
Previous spinal fusion, *n* (%)	7 (30.4)	11 (33.3)	0.995
Iliac fixation, *n* (%)	12 (52.2)	18 (54.5)	0.997
Muscular quantity of T12-L1 (CSA of erector spinae), cm^2^	23.8 ± 4.2	32.2 ± 10.1	<0.001 *
Muscular quality of T12-L1 (Fat infiltration in CSA of erector spinae)			<0.001 *
Mild to moderate (<50%), *n*	4	21	
Severe (≥50%), *n*	13	3	
Disc degeneration of T12-L1			0.923
Mild to Moderate (Pfirrmann grade 1~3)	7	12	
Severe (Pfirrmann grade 4 and 5)	10	12	

BMI, body mass index; BMD, bone mineral density; ASA, American Society of Anesthesiologists; CSA, cross-sectional area. Measurement of muscularity and disc degeneration of T12-L1 was conducted using axial view of T2 weighted magnetic resonance (MR) images of T12-L1 level. *, statistically significant if *p* < 0.05.

**Table 4:** Univariate analysis of radiographic risk factors (spinopelvic parameters).

Risk Factors	PJK Group (*n* = 23)	Non-PJK Group (*n* = 33)	*p*
Preoperative Parameters			
TK (°), mean ± SD	7.6 ± 14.3	13.9 ± 12.7	0.153
TLK (°), mean ± SD	0.75 ± 0.9	0.67 ± 1.0	0.082
PJA (°), mean ± SD	2.98 ± 8.0	1.99 ± 12.2	0.583
LL (°), mean ± SD	7.2 ± 24.7	20.2 ± 24.5	0.115
SS (°), mean ± SD	23.0 ± 8.5	30.1 ± 12.3	0.057
PT (°), mean ± SD	34.0 ± 7.2	25.8 ± 13.0	0.032 *
PI (°), mean ± SD	56.9 ± 7.0	55.5 ± 10.9	0.663
C7 SVA (mm), mean ± SD	92.0 ± 48.5	85.1 ± 43.4	0.720
Immediate (3 months) postoperative parameters			
TK (°), mean ± SD	20.7 ± 8.4	19.9 ± 17.3	0.876
TLK (°), mean ± SD	23.5 ± 9.9	8.7 ± 12.2	<0.001 *
PJA (°), mean ± SD	20.9 ± 7.7	10.7 ± 8.1	<0.001 *
LL (°), mean ± SD	43.8 ± 13.5	45.9 ± 9.4	0.569
SS (°), mean ± SD	32.0 ± 9.4	35.6 ± 8.1	0.215
PT (°), mean ± SD	24.1 ± 11.2	18.7 ± 8.6	0.092
PI (°), mean ± SD	55.8 ± 7.4	53.7 ± 7.0	0.376
C7 SVA (mm), mean ± SD	49.9 ± 38.9	37.3 ± 35.6	0.303
Changes between preoperative and immediate postoperative parameters			
ΔTK (°), mean ± SD	−13.1 ± 11.5	−5.8 ± 22.1	0.231
ΔLL (°), mean ± SD	36.6 ± 27.7	24.8 ± 27.2	0.180
ΔSS (°), mean ± SD	8.9 ± 10.8	5.2 ± 9.8	0.249
ΔPT (°), mean ± SD	−9.9 ± 12.2	−6.8 ± 8.3	0.338
ΔC7 SVA (mm), mean ± SD	−42.1 ± 56.8	−47.7 ± 45.9	0.724

TK, thoracic kyphosis; TLK, thoracolumbar kyphosis; PJA, proximal junctional angle; LL, lumbar lordosis; SS, sacral slope; PT, pelvic tilt; PI, pelvic incidence; SVA, sagittal vertical axis; *, statistically significant if *p* < 0.05.

**Table 5:** Logistic regression analysis of potential risk factors associated with PJK following L1-Sacrum fusion.

Parameters	*p*	OR	95% CI
Female sex	0.167		
Osteoporosis	0.021 *	20.50	1.59–264.99
BMI (kg/m^2^)	0.620		
Muscle quantity of T12-L1 (CSA of erector spinae)	0.082		
Muscle quality of T12-L1 (Fat infiltration in CSA of erector spinae)	0.002 *	43.60	4.11–463.06
Preoperative PT (°)	0.852		
Immediate postoperatve TLK (°)	0.161		
Immediate postoperatve PJA (°)	0.124		

OR, odds ratio; BMI, body mass index; CSA, cross-sectional area; PT, pelvic tilt; TLK, thoracolumbar kyphosis; PJA, proximal junctional angle; CI, confidence interval; * statistically significant if *p* < 0.05.

## Data Availability

The patients’ data were collected in Kyung Hee University Hospital at Gangdong. The datasets generated and/or analyzed during the current study are available from the corresponding author (KYH) on reasonable request.

## Abbreviations

PJK	Proximal junctional kyphosis
PJA	Proximal junctional angle
ASD	Adult spinal deformity
TK	Thoracic kyphosis
TLK	Thoracolumbar kyphosis
LL	Lumbar lordosis
PT	Pelvic tilt
SS	Sacral slope
PI	Pelvic incidence
SVA	Sagittal vertical axis
BMI	Body mass index
BMD	Bone mineral density
ASA	American Society of Anesthesiologists
CSA	Cross-sectional area
MR	Magnetic resonance
SRS	Scoliosis Research Society
ODI	Oswestry disability index
